# Analysis of Dynamic Stability Control of Light Source in Immersion DUV Lithography

**DOI:** 10.3390/mi16111207

**Published:** 2025-10-23

**Authors:** Yihua Zhu, Dandan Han, Chuang Wu, Sen Deng, Yayi Wei

**Affiliations:** 1 School of Integrated Circuits, University of Chinese Academy of Sciences, Beijing 101408, China; 2 Institute of Microelectronics of the Chinese Academy of Sciences, Beijing 100029, China

**Keywords:** high NA, DUV lithography, excimer laser, dynamic stability, NILS

## Abstract

Immersion deep ultraviolet (DUV) lithography remains an indispensable core technology in advanced integrated circuit manufacturing, particularly when combined with multiple patterning techniques to achieve sub-10 nm feature patterning. However, at advanced technology nodes, dynamic instabilities of DUV light sources—including spectral characteristics (bandwidth fluctuations, and center wavelength drift), coherence variations, and pulse-to-pulse energy instability—can adversely affect imaging contrast, normalized image log-slope (NILS), and critical dimension (CD) uniformity. To quantitatively assess the impact of laser parameter fluctuations on NILS and CD, this work establishes systematic physical models for imaging perturbations caused by multi-parameter laser output instabilities under immersion DUV lithography. Through simulations, we evaluate the influence of laser parameter variations on the imaging fidelity of representative line/space (L/S) and tip-to-line (T2L) structures, thereby validating the proposed perturbation model. Research demonstrates that the spectral attributes (bandwidth fluctuation and center wavelength drift), coherence variations, and pulse energy instability collectively induce non-uniform electric field intensity distribution within photoresist, degrading NILS, and amplifying CD variation, which ultimately compromise pattern fidelity and chip yield. Notably, at advanced nodes, pulse energy fluctuation exerts a significantly greater influence on imaging errors compared to bandwidth and wavelength variations. To satisfy the 10% process window requirement for 45 nm linewidths, pulse energy fluctuations should be rigorously confined within 1%. This research provides theoretical foundations and practical insights for the design of dynamic stability control of light source and process optimization of next-generation DUV light sources.

## 1. Introduction

Driven by process maturity and cost-effectiveness, 193 nm ArF immersion lithography continues to serve as a pivotal manufacturing solution for logic devices (e.g., FinFET/GAA architectures) and high-density memory (including DRAM and 3D NAND) at 5–28 nm technology nodes [1,2,3]. As device dimensions approach fundamental physical limits, the dynamic stability of light source performance has emerged as a critical factor influencing the final pattern quality. At these advanced nodes, minute fluctuations originating from the light source can be significantly amplified through the intricate optical system, inducing increased line edge roughness and resolution degradation that directly compromise patterning fidelity.

Within lithographic scanner systems, the DUV light source—typically an ArF excimer laser—plays a significant role in determining image contrast and critical dimension (CD). Acting as the optical propagation origin, the source system delivers laser beams with sufficient energy and stable spectral characteristics to meet the stringent requirements of modern lithography. ArF excimer lasers (λ = 193 nm), the predominant DUV source, are characterized by narrow spectral linewidths (mainstream E95 ≈ 0.3 pm), high peak powers (typically 60–90 W), and short pulse widths [4,5,6,7]. Compared with extreme-ultraviolet (EUV) lithography, which has a complex process for generating high-energy photons, the DUV platform has a higher level of system maturity. Nevertheless, as the industry moves toward higher numerical apertures (e.g., 1.35 NA immersion systems) and tighter overlay budgets, imaging performance becomes markedly more sensitive to fluctuations in source parameters. Dynamic instabilities—including bandwidth fluctuation, center wavelength drift, and pulse energy variation—can propagate through the complex optical path system to the focal plane, inducing non-uniform exposure dose distribution that manifests as CD errors, pattern shift, etc., causing the lithography results to deviate from the expectation [8,9,10].

Although advanced monitoring and active feedback schemes for source parameters [11,12] have significantly enhanced source stability, dynamic output fluctuations persist as a critical constraint on imaging quality in high-volume manufacturing (HVM) environments. The evolution of resolution enhancement technologies (RETs)—such as optical proximity correction (OPC) [13,14,15], source-mask optimization (SMO), multi-pole illumination, and inverse lithography technology (ILT) [16,17,18,19,20]—has further heightened sensitivity to source instabilities. In these contexts, even minute source variations can induce illumination field non-uniformity, degrading the normalized image log-slope (NILS) and exacerbating CD variations, ultimately reducing pattern fidelity and yield. Consequently, systematic investigation of dynamic source behavior and its impact on imaging robustness has become a critical research focus for improving DUV lithographic performance at advanced nodes.

Previous studies have extensively explored parameter control in DUV source systems [21,22,23,24,25,26]. Prior research [21] demonstrated that spectral parameter variations (E95% bandwidth and spectral shape) critically affect pattern displacement at forbidden Pitches in sub-32 nm logic nodes. In ref. [22], the researchers established numerical models correlating wavelength drift with chromatic aberrations and evaluated lens sensitivity to wavelength shifts in KrF systems. Temporal and spatial coherence-induced dynamic speckle effects were analytically modeled in ref. [23], revealing that coherence contributes less significantly to linewidth roughness (LWR) than defocus-induced errors. Ref. [24] reported development of an ArF immersion XLR laser system that increased pulse energy from 10–15 mJ to 15–20 mJ to achieve ~120 W high-power output, and validated its stability under various operating conditions. However, existing studies predominantly focus on static parameter optimization (center wavelength *λ*_0_, bandwidth Δ*λ*, etc.), lacking quantified analysis of how dynamic instabilities—bandwidth fluctuation, wavelength drift, and pulse energy variation—impact imaging at advanced nodes. Additionally, prior models primarily address ≥14 nm nodes, providing limited guidance for contemporary lithography processes.

**Figure 1:**
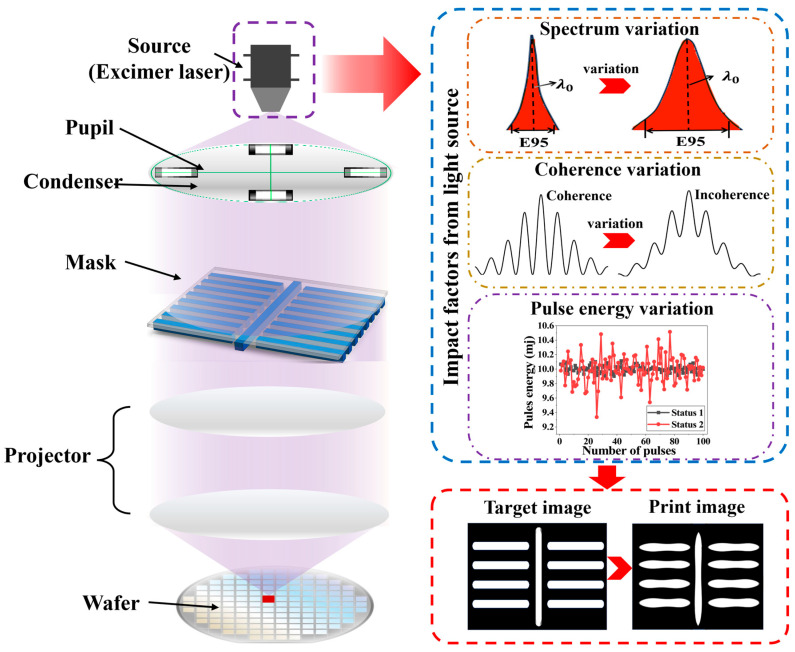
Schematic diagram of lithography imaging affected by changes in the stability of main parameters (spectral characteristics, coherence, and pulse energy) of the light source system.

This study aims to systematically analyze the dynamic stability of DUV light sources from a statistical perspective, specifically investigating how instability modes (spectrum, coherence, and pulse energy variation, as shown in Figure 1) degrade imaging performance under a practical lithographic background. We establish a physics-based modeling approach, clarify the transmission path of light source fluctuations in the imaging process, and quantify exposure dose errors arising from spectral, coherence, and pulse energy instabilities. Furthermore, realistic parameter stability boundaries are explored via simulation, providing critical design guidelines and optimization pathways for next-generation high-stability lithography platforms.

## 2. Theoretical Model

### 2.1. Lithographic Imaging Model

**Figure 2:**
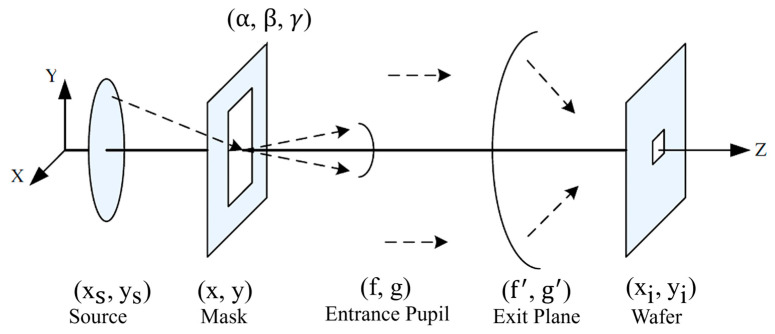
Schematic diagram of incident light propagation in the lithography system [25].

As illustrated in Figure 2, planar waves emitted from the excimer light source irradiate the photomask. These waves undergo diffraction by mask patterns, generating multiple planar wavefronts that propagate in distinct directions. Diffracted orders captured within the numerical aperture (NA) enter the projection lens via the entrance pupil. After transmission through the projection optics to the exit pupil, these diffraction orders generate planar waves that propagate to the image plane, where they interfere to form the aerial image. Under partially coherent illumination, the effective source can be decomposed into an ensemble of spatially incoherent point sources. No interference occurs between wavefronts generated by distinct point sources. Following the principle of partial coherence integration, the total image plane intensity distribution is obtained by computing the intensity contributions from each point source and summing them [27,28,29]:

(1) $$I({\hat{x}}_{i},{\hat{y}}_{i}\;)=\int_{-\infty }^{+\infty }\begin{array}{c} ∭TCC({\hat{f}}^{\prime },{\hat{g}}^{\prime };{\hat{f}}^{\prime \prime },{\hat{g}}^{\prime \prime })M({\hat{f}}^{\prime },{\hat{g}}^{\prime })M^{*}({\hat{f}}^{\prime \prime },{\hat{g}}^{\prime \prime })\\ \exp\{-j2\pi [{(\hat{f}}^{\prime }-{\hat{f}}^{\prime \prime }){\hat{x}}_{i}+({\hat{g}}^{\prime }-{\hat{g}}^{\prime \prime }){\hat{y}}_{i}]\}d{\hat{f}}^{\prime }d{\hat{g}}^{\prime }d{\hat{f}}^{\prime \prime }d{\hat{g}}^{\prime \prime } \end{array}$$

(2) $$TCC({\hat{f}}^{\prime },{\hat{g}}^{\prime };{\hat{f}}^{\prime \prime },{\hat{g}}^{\prime \prime })=\iint_{-\infty }^{+\infty }P(\hat{f},\hat{g})\;H(\hat{f}+{\hat{f}}^{\prime },\hat{g}+{\hat{g}}^{\prime })H^{*}(\hat{f}+{\hat{f}}^{\prime \prime },\hat{g}+{\hat{g}}^{\prime \prime })\;d\hat{f}d\hat{g}$$

where *j* denotes the imaginary unit; $\left({\hat{x}}_{i},{\hat{y}}_{i}\;\right)$ are normalized spatial coordinates in the image plane; $\left(\hat{f},\hat{g}\right)$ and $\left({\hat{f}}^{\prime },{\hat{g}}^{\prime }\right)$ represent normalized spatial frequency coordinates in the pupil plane and diffracted mask spectrum, respectively; and $M({\hat{f}}^{\prime },{\hat{g}}^{\prime })$ is the Fourier transform of the mask pattern. Under the Kirchhoff approximation for thin mask diffraction, the mask’s spectral amplitude and phase remain translation-invariant for incident planar waves. This decouples the mask function from the optical system, with the latter described by the Transmission Cross Coefficients (TCCs). Here, $H(\hat{f},\hat{g})$ represents the projection lens transfer function:

(3) $$H(\hat{f},\hat{g})=\{\begin{array}{c} R(\hat{f},\hat{g})T(\hat{f},\hat{g})\exp\left[-j\frac{2\pi }{\lambda }W(\hat{f},\hat{g})\right],\;\;\;\;\;\sqrt{{\hat{f}}^{2}+{\hat{g}}^{2}}\leq 1\\ \;\;\;\;\;\;\;\;\;\;\;\;\;\;\;\;\;\;\;\;\;\;\;\;\;\;\;\;\;\;\;\;0,\;\;\;\;\;\;\;\;\;\;\;\;\;\;\;\;\;\;\;\;\;\;\;\;\;\;\;\;\;\;\;\;\;\;\;\;\;\;otherwise\;\;\;\;\;\;\;\; \end{array}$$

This equation encompasses NA, obliquity factor R, and wavefront aberration W, collectively modulating diffracted mask orders. $P(\hat{f},\hat{g})$ denotes the effective source intensity distribution. For conventional circular illumination with a partial coherence factor (defined as the ratio of source radius to objective pupil radius):

(4) $$P(\hat{f},\hat{g})=\frac{1}{\pi \sigma^{2}}circ(\frac{\sqrt{{\hat{f}}^{2}+{\hat{g}}^{2}}}{\sigma })=\{\begin{array}{c} \frac{1}{\pi \sigma^{2}},\;\;\;\;\;\;\;\;\;\;\;\;\;\;\;\sqrt{{\hat{f}}^{2}+{\hat{g}}^{2}}\leq \sigma \\ 0,\;\;\;\;\;\;\;\;\;\;\;\;\;\;\;\;\;\;\;otherwise\;\;\; \end{array}$$

Equations (1)–(4) describe lithographic imaging under monochromatic conditions. From these expressions, it is evident that the imaging outcomes in lithography systems are influenced by multiple components, including the light source, illumination system, mask, and projection system.

In a projection lithography, different wavelengths produce different focal planes. A finite spectral bandwidth may be regarded as an ensemble of monochromatic components at different wavelengths. The superposition of these monochromatic contributions affects the system focus and leads to image blurring and a reduction in resolution. In bandwidth effect research [23,24], the finite-bandwidth image intensity $I_{image}({\hat{x}}_{i},{\hat{y}}_{i})$ is commonly modeled as the integral of monochromatic image intensities $I_{\lambda_{0}}({\hat{x}}_{i},{\hat{y}}_{i};\;\lambda )$ evaluated at multiple planes of defocus, i.e.,

(5) $$I_{image}({\hat{x}}_{i},{\hat{y}}_{i})=\int I_{\lambda_{0}}({\hat{x}}_{i},{\hat{y}}_{i};\;\lambda )\cdot S(\lambda )d\lambda$$

where S(λ) is the spectral shape of the output laser. The spectral shape S(λ) is typically well approximated by a Gaussian function, for example:

(6) $$S_{gauss}(\lambda )=\frac{1}{\sqrt{2\pi }v}\exp\left(\frac{{\left(\lambda -\lambda_{0}\right)}^{2}}{2v^{2}}\right),\;v=\frac{∆\lambda }{2\sqrt{2\ln2}}$$

where *λ*_0_ is the nominal central wavelength and ∆*λ* characterizes the spectral width.

### 2.2. Impact of Light Source System Stability on Lithographic Imaging

#### 2.2.1. Analysis of Spectral Characteristic Stability Variations

Wavelength and bandwidth stability constitute critical attributes of pulsed lasers. Owing to the pulse emission nature of excimer lasers, consistency between laser pulses cannot be guaranteed. Research indicates [23,24] that fluctuations in laser output parameters (e.g., wavelength and bandwidth) predominantly follow a normal distribution. Consequently, parameters of the normal distribution $\left(\mu ,\;\sigma^{2}\right)$ can characterize light source system stability. Assuming wavelength and bandwidth stability variations adhere to independent normal distributions: $\lambda \sim N(\mu_{\lambda },\;\sigma_{\lambda }^{2});\Delta \lambda \sim N(\mu_{∆\lambda },\;\sigma_{\Delta \lambda }^{2})$.

According to the finite-bandwidth lithographic imaging formulation (Equations (5) and (6)), variations in spectral characteristics (wavelength *λ* and bandwidth Δ*λ*) induce alterations in light intensity, generating a sequence of intensity distributions (*I*_1_, …, *I_n_*). The temporal accumulation of intensity forms the exposure dose $D=\int_{0}^{t}I(t)dt$. Variations in intensity distribution cause fluctuations in received exposure dose on the wafer, thereby affecting lithographic imaging. In DUV systems, the exposure dose relates to the scanner velocity *V* and the number of received pulses *N*, and may be summarized as

(7) $$D_{scan}(x,y)=\sum_{n=1}^{N}\frac{1}{V(y_{n})}\int I_{n}(x,y-y_{n})dy_{n}$$

where *y_n_* is the scan position for the *n*th pulse. For analytical simplicity we assume a constant scan speed *V*, then the accumulated dose at a spatial point *(x, y)* can be approximated by

(8) $$D_{scan}(x,y)\approx \frac{1}{V}\sum_{n=1}^{N}\bar{I_{n}}$$

Here, we assume the average intensity $\bar{I_{t}}$ at the point under nominal conditions $\left(\lambda_{t},\;{∆\lambda }_{t}\right)$ accumulates target dose *D_t_*. Dynamic spectral variations (*λ_n_*, Δ*λ_n_*) cause intensity variations (*I*_1_, …, *I_n_*), leading to dynamic exposure doses (*D*_1_, …, *D_m_*). Owing to dynamic spectral variations, the instantaneous intensity sequence becomes (*I*_1_, …, *I_n_*) and the actual doses received on the wafer form a stochastic sequence (*D*_1_, …, *D_m_*). The dose accumulated as a function of the spectral variations (*λ_n_*, Δ*λ_n_*) may be written as

(9) $$D_{\lambda }=\frac{1}{V}\sum_{n=1}^{N}\bar{I_{n}}(\lambda_{n},\;{\Delta \lambda }_{n})$$

Since *λ_n_* and Δ*λ_n_* are independent and identically distributed random variables, $\bar{I_{n}}$ is also stochastic. According to the probability theory, the variance of *D_λ_* can be expressed as

(10) $$Var(D_{\lambda })=\frac{N}{V^{2}}Var(\bar{I_{n}})$$

Under the finite-bandwidth imaging model (Equations (5) and (6)), $\bar{I_{n}}$ is generally a nonlinear function of *λ* and Δ*λ*. For small perturbations, we may use a first-order Taylor expansion about the nominal operating point $\left(\lambda_{t},{\Delta \lambda }_{t}\right)$. Denoting $\bar{I_{t}}=I(\lambda_{t},{\Delta \lambda }_{t})$ and $\delta \lambda_{n}=\lambda_{n}-\lambda_{t},\;\delta {\Delta \lambda }_{n}={\Delta \lambda }_{n}-{\Delta \lambda }_{t}$, we have Equation (11).

(11) $$\bar{I_{n}}\approx \bar{I_{t}}+\frac{\partial \bar{I}}{\partial \lambda }\delta \lambda_{n}+\frac{\partial \bar{I}}{\partial {\Delta \lambda }_{n}}\delta {\Delta \lambda }_{n}+\cdots o^{n}$$

Substituting Equation (11) into Equation (10) and retaining only first-order contributions yields the following:

(12) $$Var(D_{\lambda })\approx \frac{N}{V^{2}}[{\left(\frac{\partial \bar{I}}{\partial \lambda }\right)}^{2}\sigma_{\lambda }^{2}+{\left(\frac{\partial \bar{I}}{\partial \Delta \lambda }\right)}^{2}\sigma_{\Delta \lambda }^{2}]$$

where $\sigma_{\lambda }^{2}$ and $\sigma_{\Delta \lambda }^{2}$ are the variances of *λ*_n_ and Δ*λ*_n_, respectively. Therefore, the relative standard deviation of the exposure dose at a given wafer point due to spectral stability variations can be expressed as

(13) $$\Delta_{\lambda ,\Delta \lambda }=\frac{\sigma_{D1}}{D_{t}}\approx \frac{1}{\sqrt{N}}\frac{1}{I_{t}}\sqrt{{\left(\frac{\partial \bar{I}}{\partial \lambda }\right)}^{2}\sigma_{\lambda }^{2}+{\left(\frac{\partial \bar{I}}{\partial \Delta \lambda }\right)}^{2}\sigma_{\Delta \lambda }^{2}}$$

Equation (13) quantifies how wavelength and bandwidth stability impact dose variability on the wafer plane.

#### 2.2.2. Analysis of Coherence-Induced Imaging Effects

Optical coherence comprises temporal and spatial components. Temporal coherence quantifies the correlation of a light wave with itself at a spatial point across different times. For excimer lasers, temporal coherence inversely correlates with spectral width via τ_c_ = 1/∆ν, where τ_c_ is the coherence time and ∆ν is the spectral width in frequency units (the conversion between frequency-domain and wavelength-domain spectral widths is $\Delta \nu =\frac{c}{\lambda^{2}}\cdot ∆\lambda$). Longer τ_c_ indicates superior phase stability over time. Spatial coherence reflects correlation between different spatial points at one time [30]. Larger coherence areas imply sustained phase uniformity over broader regions. Both temporal and spatial coherence of the laser influence lithographic imaging. Higher temporal coherence (narrower spectral bandwidth) tends to produce sharper interference fringes, which is beneficial for faithful transfer of fine patterns [31,32].

As per dynamic speckle theory [23], laser coherence induces intensity inhomogeneity (speckle) at the image plane, causing dose variation. The statistics of speckle can be described by introducing a temporal degree of freedom M, defined as the ratio of the square of the expected integrated intensity per unit area to the variance of the integrated intensity. Assuming that the laser spectral shape is Gaussian, $S_{gauss}(\lambda )$, the temporal degree of freedom under this spectral model can be computed as

(14) $$M=\frac{{D_{t}}^{2}}{{\sigma_{d}}^{2}}=\sqrt{\frac{\pi^{2}}{4{\left(\ln2\right)}^{2}}{\left(\frac{c\Delta T_{FWHM}\cdot \Delta \lambda }{\lambda_{0}^{2}}\right)}^{2}+1}$$

$\Delta T_{FWHM}$ denotes the full width at half-maximum (FWHM) of the pulse temporal duration. Considering the regime where $\frac{c\Delta T_{FWHM}\cdot \Delta \lambda }{\lambda_{0}^{2}}\gg 1$, the temporal degree of freedom M can be approximated by the following expression:

(15) $$M\approx \frac{\pi }{2\ln2}\cdot \frac{c\Delta T_{FWHM}\cdot \Delta \lambda }{\lambda_{0}^{2}}$$

Relative dose fluctuation due to coherence is

(16) $$\frac{\sigma_{d}}{D_{t}}=\frac{1}{\sqrt{M}}\approx \sqrt{\frac{2\ln2}{\pi }}\cdot \frac{\lambda_{0}}{\sqrt{c\Delta T_{FWHM}\cdot \Delta \lambda }}$$

From Equation (16), it follows that $\frac{\sigma_{d}}{D_{t}}\propto \frac{\lambda_{0}}{\sqrt{\Delta \lambda }}\cdot \frac{1}{\sqrt{\Delta T_{FWHM}}}$, which indicates that, under static conditions (i.e., when the output spectral characteristics of the source remain constant), the dose fluctuations induced by source coherence are inversely related to the spectral bandwidth. In this regime, increasing the bandwidth effectively reduces the amplitude of dose fluctuations caused by source coherence.

Dynamic spectral variation ($\lambda \sim N(\mu_{\lambda },\;\sigma_{\lambda }^{2}),\Delta \lambda \sim N(\mu_{∆\lambda },\;\sigma_{\Delta \lambda }^{2})$) simultaneously changes speckle statistics *M*, altering coherence-driven dose (*D*_1_, …, *D_m_*) _coherence_. By analyzing the statistical properties of the coherence distribution (*D*_1_, …, *D_m_*) _coherence_, we can extract the characteristic dose-variation signatures induced by source coherence under dynamic source behavior. Equation (17) can be written as

(17) $$O(\Delta \lambda )=\frac{1}{\sqrt{M(\Delta \lambda )}},\;M(\Delta \lambda )\approx K\cdot \Delta \lambda ,\;K=\frac{\pi }{2\ln2}\cdot \frac{c∆T_{FWHM}}{\lambda^{2}}$$

Therefore, the variance $Var(D_{c})$ can be reduced to the problem of determining the variance of the function $O=K^{-1/2}\cdot x^{-1/2}$. According to the statistical formula, we can derive the expression for $Var(D_{c})$ (the detailed algebraic derivation is provided in Appendix A):

(18) $$Var(O)=\frac{1}{4}s_{0}^{2}{\left(\frac{\sigma_{\Delta \lambda }}{\mu_{\Delta \lambda }}\right)}^{2},\;s_{0}=\sqrt{\frac{2\ln2}{\pi }}\frac{\lambda }{\sqrt{c\Delta T\mu_{\Delta \lambda }}}$$

(19) $${∆}_{c}=\frac{\sigma_{D_{c}}}{D_{c}}=\frac{s_{0}}{2}\cdot \frac{\sigma_{\Delta \lambda }}{\mu_{\Delta \lambda }}$$

#### 2.2.3. Analysis of Pulse Energy Stability-Induced Imaging Effects

ArF excimer lasers emit not as a continuous wave but as a series of short pulses. Each pulse is produced by a rapid, transient release of energy within the laser. Individual pulses have high energy density and can deliver the required dose to the photoresist in a very short time. These pulses are typically organized into sequences known as “Bursts.” A “Burst” sequence contains a fixed number of pulses that are emitted consecutively at a specified repetition frequency. The “Burst” mode is designed to optimize energy distribution and thermal management while maintaining beam quality and required output characteristics for various lithographic steps [6].

This operating mode enables efficient energy management and heat control while preserving output energy and beam quality. The laser energy parameters relevant to this mode include average power, repetition frequency, and single-pulse energy. The average power is the time-averaged energy output of the laser during continuous operation, and it directly affects throughput: higher average power permits delivery of sufficient exposure energy in less time, increasing productivity, but excessive average power risks over-exposure and degraded imaging fidelity. For a fixed total energy output over a given time interval, the single-pulse energy can be adjusted by changing the pulse repetition frequency. Consequently, the single-pulse energy and its stability directly determine the stability of the delivered exposure dose and strongly influence the post-exposure pattern accuracy.

The exposure dose per unit area on the wafer can be expressed as the total energy contributed by the N pulses used to expose the slit (from the jth pulse to the (N + j − 1)th pulse) divided by the exposed area *A*, i.e.,

(20) $$D_{p}=\frac{1}{A}\sum_{i=j}^{N+j\;-\;1}E_{i}$$

Assume the single-pulse energies are independent and identically distributed random variables, $E_{pluse}\sim (\mu_{E},\sigma_{E}^{2})$, and the variation of exposure doses due to pulse energy fluctuations is *(*$D_{p1}$, …, $D_{\mathrm{pn}}$*)*. The variance $Var(D_{p})$ is

(21) $$Var(D_{p})=\frac{1}{A}\sum_{i=j}^{N+j\;-\;1}Var(E_{i})=\frac{N}{A}.\sigma_{E}^{2}$$

Then, the dose fluctuation caused by pulse dose stability can be expressed as

(22) $$\Delta_{p}=\frac{\sigma_{D_{p}}}{D_{t}}=\sqrt{\frac{1}{A\cdot N}}\cdot \frac{\sigma_{E}}{\mu_{E}}$$

Finally, if we assume that the three instability mechanisms treated in this work—(i) spectral characteristic fluctuations, (ii) coherence-induced speckle variability, and (iii) pulse energy fluctuations—are mutually independent, then the total dose variability at a given wafer location can be obtained by summing the contributions in variance (or equivalently combining relative fluctuations in quadrature). Denoting the relative standard deviations due to spectral effects, coherence, and pulse energy by $\Delta_{\lambda ,\Delta \lambda }$, ${∆}_{c}$, and $\Delta_{p}$, respectively, the overall relative dose fluctuation is

(23) $${∆}_{total}^{2}=\frac{1}{N\cdot I_{t}^{2}}[{\left(\frac{\partial \bar{I}}{\partial \lambda }\right)}^{2}\sigma_{\lambda }^{2}+{\left(\frac{\partial \bar{I}}{\partial \Delta \lambda }\right)}^{2}\sigma_{\Delta \lambda }^{2}]+\frac{1}{4}s_{0}^{2}{\left(\frac{\sigma_{\Delta \lambda }}{\mu_{\Delta \lambda }}\right)}^{2}+\frac{1}{A\cdot N}\cdot {\left(\frac{\sigma_{E}}{\mu_{E}}\right)}^{2}$$

## 3. Results and Discussion

### 3.1. Simulation Scheme Design

We consider the operating conditions of the excimer light source in an immersion DUV scanner in normal operation. The excimer laser operates in pulsed mode (typical repetition rate: 6 kHz). The spatial information encoded on the mask is carried by each pulse, relayed through the optical system, and projected onto the wafer via a dynamically scanned slit. Pattern transfer occurs when the accumulated dose per unit area reaches the threshold of the resist. During this process, variations in the source-system stability lead to pulse-to-pulse changes in the emitted laser characteristics. As established in Section 2, key output parameters—bandwidth, wavelength, and pulse energy stability—are modeled as normally distributed random variables described by μ and σ to emulate realistic laser output conditions in production. Rigorous lithography simulations were carried out using the commercial simulator S-Litho™ (Version 2024.09).

(1) Exposure Settings and Test Patterns

Table 1 summarizes pertinent parameters for the DUV lithography simulation. Test mask patterns include representative one-dimensional line/space (L/S) and two-dimensional tip-to-line (T2L) structures from integrated circuit metal interconnect layers. Multiple stochastic simulations were executed for each spectral distribution (*μ*, *σ*). Image quality was evaluated using two metrics: CD error (the absolute deviation of CD under perturbed source conditions from the CD under nominal conditions) and the normalized image log-slope (NILS). Line-edge/width roughness (LER/LWR) was not considered. This study exclusively examines image shift, NILS, and CD variation induced by source stability fluctuations.

(2) Laser Stability Parameters

Reference nominal operating values for the excimer laser were chosen as a spectral bandwidth of 300 fm (≈0.3 pm) and a center wavelength of 193.0 nm. These nominal values (μ) serve as the baseline and a set of stability parameter variations (σ)—including bandwidth changes, wavelength drifts and pulse energy fluctuations—was defined to study their effects on exposure dose variation and imaging performance. The detailed laser parameter stability conditions are listed in Table 2. Because this work focuses on source-system stability, all variables other than the source parameters were held ideal and constant.

### 3.2. Simulation and Analysis

#### 3.2.1. Impact of Spectral Parameter Fluctuations on Aerial Images

**Figure 3:**
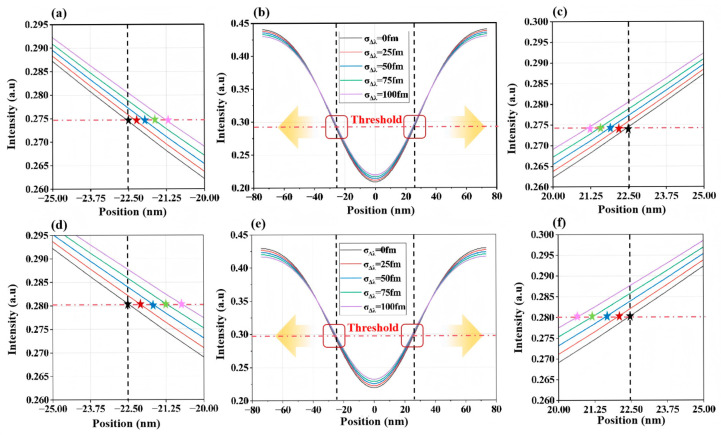
Two-dimensional aerial image intensity variations induced by spectral bandwidth fluctuations for L/S structures with CD = 45 nm and Pitch = 150 nm. (**a**–**c**): Δ*λ* = 200 fm, (**d**–**f**): Δ*λ* = 300 fm. The black dashed lines denote the two ends of the line structures on the mask pattern, while the red dashed lines indicate the intensity threshold corresponding to a printed linewidth of 45 nm after exposure. The transparent yellow arrows point to the magnified regions marked by the red rectangles in panels (**b**) or (**e**), which are shown in panels (**a**), (**c**), or (**d**), (**f**), respectively. In panels (**a**), (**c**), or (**d**), (**f**), the star symbols in different colors indicate the actual positions of the two ends of the line structures at the intensity threshold.

**Figure 4:**
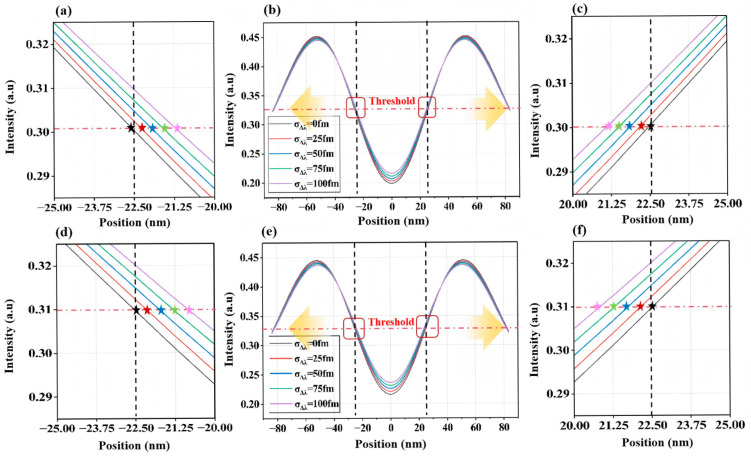
Two-dimensional aerial image intensity variations induced by spectral bandwidth fluctuations for T2L structures with CD = 45 nm and Pitch = 150 nm. (**a**–**c**): Δ*λ* = 200 fm, (**d**–**f**): Δ*λ* = 300 fm. The black dashed lines denote the two ends of the line structures on the mask pattern, while the red dashed lines indicate the intensity threshold corresponding to a printed linewidth of 45 nm after exposure. The transparent yellow arrows point to the magnified regions marked by the red rectangles in panels (**b**) or (**e**), which are shown in panels (**a**), (**c**), or (**d**), (**f**), respectively. In panels (**a**), (**c**), or (**d**), (**f**), the star symbols in different colors indicate the actual positions of the two ends of the line structures at the intensity threshold.

Equations (5) and (6) demonstrate that variations in spectral characteristics (wavelength *λ*, and bandwidth ∆λ) alter intensity distribution *I* (*x*, *y*) on the wafer. Figure 3 and Figure 4 illustrate the relationship between 2D aerial image intensity distribution and $\sigma_{\Delta \lambda }$ changes for two representative structures (L/S and T2L) under different bandwidths. When using the intensity threshold under nominal spectral conditions ($\lambda =\lambda_{t},\;{\Delta \lambda =∆\lambda }_{t}$, and  = 0 fm) as the exposure threshold, $\sigma_{\Delta \lambda }$ changes induce CD variations. Here, ΔCD shows a positive correlation with $\sigma_{\Delta \lambda }$, confirming the influence of laser spectral instability on lithographic imaging. Comparing intensity distributions under baseline bandwidths (∆λ = 200 fm vs. 300 fm), bandwidth stability variations at Δ*λ* = 300 fm exert a stronger impact on aerial image intensity than those at Δ*λ* = 200 fm.

**Figure 5:**
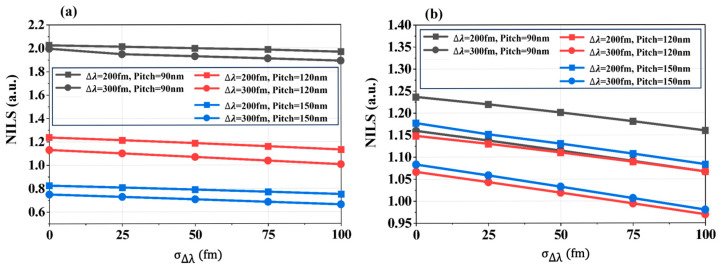
NILS variations caused by spectral bandwidth fluctuations. (**a**) NILS for L/S patterns at dense (90 nm), semi-dense (120 nm), and sparse (150 nm) Pitches; (**b**) NILS for T2L patterns at dense (90 nm), semi-dense (120 nm), and sparse (150 nm) Pitches.

In lithographic imaging, the normalized image log-slope (NILS = *w* dln*I*/d*x*, where *w* is the normalization coefficient) quantifies the steepness of the image edge transition from dark to bright. Larger NILS indicates a steeper dark-to-bright transition and hence sharper edge definition. Figure 5 presents extracted NILS values from 2D intensity distributions for dense, semi-dense, and sparse patterns of L/S and T2L structures. As bandwidth fluctuations increase (higher $\sigma_{\Delta \lambda }$), NILS monotonically decreases, degrading edge exposure fidelity and blurring the image. While NILS magnitudes differ across patterns, the declining trend remains consistent.

**Figure 6:**
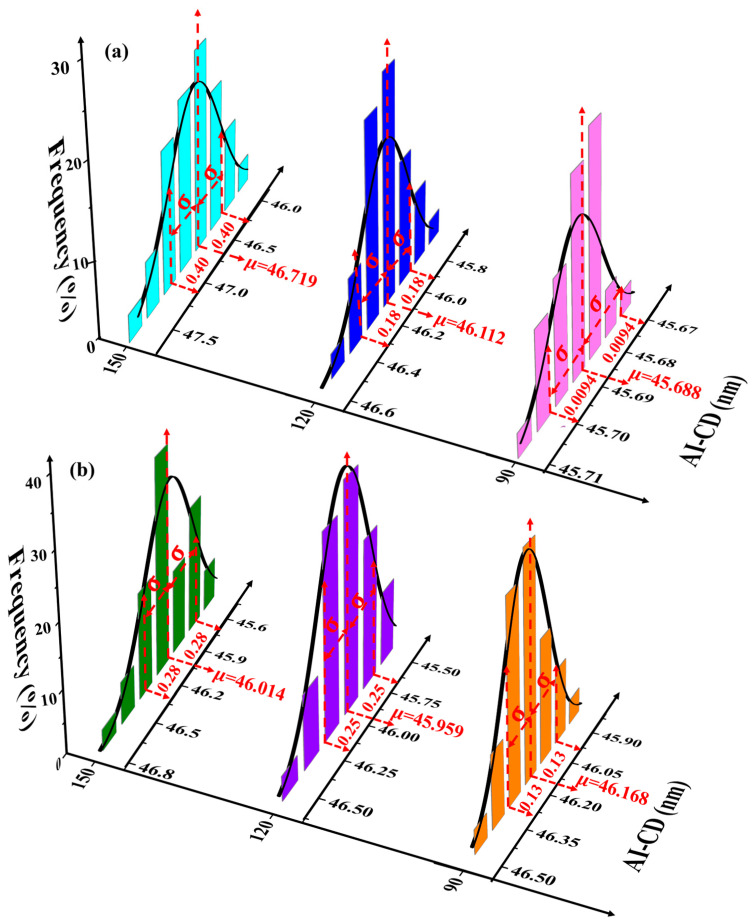
AI-CD distributions under Δ*λ* = 300 fm and $\sigma_{\Delta \lambda }$ = 100 fm. (**a**) L/S patterns (Pitches: 90/120/150 nm); (**b**) T2L patterns (Gap = 60 nm, Pitches: 90/120/150 nm).

**Figure 7:**
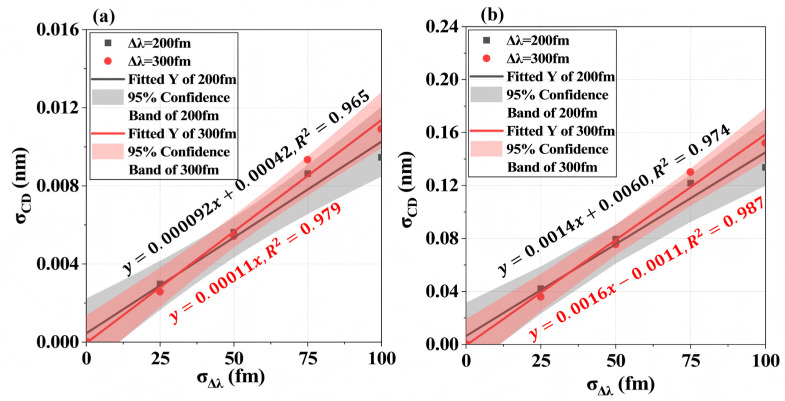
AI-CD variation induced by different spectral distributions. (**a**) L/S pattern (Pitch = 90 nm); (**b**) T2L pattern (Gap = 60 nm, Pitches = 90 nm).

From a large set of simulations, we obtained distributions of the simulated aerial-image critical dimension (AI-CD) and characterized these distributions by normal statistics (μ, σ). Figure 6 shows representative AI-CD distributions for both test patterns under Δ*λ* = 300 fm and $\sigma_{\Delta \lambda }$ = 100 fm. For sparse line structures (Pitch = 150 nm), CD fluctuations ($\sigma_{CD}$) caused by spectral instabilities significantly exceed those in dense/semi-dense patterns. Figure 7 plots the relationship between $\sigma_{CD}$ and $\sigma_{\Delta \lambda }$ for dense patterns (Pitch = 90 nm) with linear fitting. It reveals that worsening bandwidth stability increases CD variations in both L/S and T2L structures, with an approximately linear relationship $\sigma_{\Delta \lambda }$ ∝ $\sigma_{CD}$. At the dense Pitch (Pitch = 90 nm), 2D patterns ($\sigma_{CD}$ = 0.13 nm) exhibit greater sensitivity than 1D lines ($\sigma_{CD}$ = 0.0094 nm). Comparing Figure 6a,b, L/S structures show larger CD variation amplification ($\sigma_{CD}$: 0.0094 → 0.40 nm) than T2L structures ($\sigma_{CD}$: 0.13 → 0.28 nm) when transitioning from dense (Pitch = 90 nm) to sparse (Pitch = 150 nm) patterns. This indicates that pattern topology and density substantially change the lithographic performance sensitivity to spectral stability fluctuations.

#### 3.2.2. Impact of Pulse Stability on Lithographic Results

**Figure 8:**
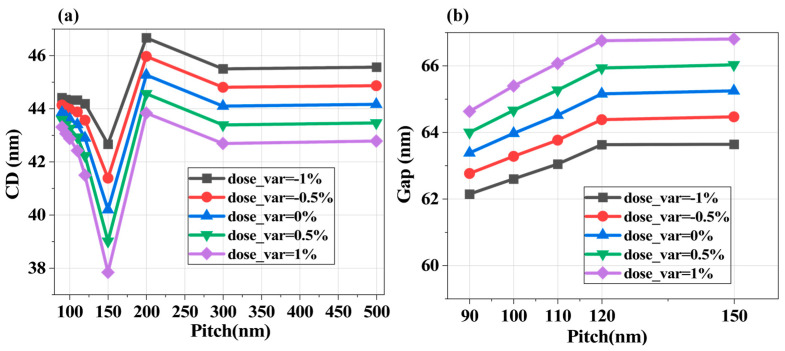
Relationship between ADI-CD and dose changes (caused by pulse energy variation) for different pattern geometries (Δ*λ* = 300 fm, $\sigma_{\Delta \lambda }$ = 100 fm). (**a**) The line structures of L/S; (**b**) the Gap structures of T2L.

Fluctuations in single-pulse energy during exposure alter the dose delivered to the resist, affecting pattern linewidth (ADI-CD). Figure 8 illustrates ADI-CD variations under dose changes for L/S and T2L mask structures. As shown in Figure 8a, without applying OPC, pulse energy fluctuations are affected by the pattern structure: ADI-CD variations in dense structures (Pitch < 110 nm) are significantly smaller than in sparse patterns. For a 45 nm linewidth with Pitch = 150 nm, a 0.5% pulse energy change induces ~1 nm ADI-CD shift. For 2D T2L structures in the Pitch < 150 nm range, Gap variations (Figure 8b) remain consistent (~0.7 nm) under pulse energy changes.

**Figure 9:**
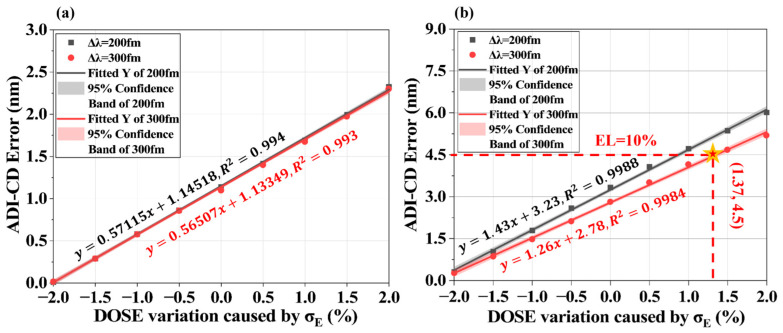
ADI-CD error induced by dose fluctuations at Pitch = 90 nm ($\mu_{E}$ = 102%, $\sigma_{E}$ = 2%). (**a**): L/S; (**b**): T2L. In panel (**b**), the star markers indicate the allowable exposure dose variation corresponding to an exposure latitude of 10%.

To quantify the contribution of pulse energy stability to CD error, we fitted the simulated data with linear regressions and obtained excellent fits (R^2^ ≈ 0.99), as plotted in Figure 9. The results show an approximately linear dependence of ADI-CD on pulse energy variation. Under the simulated conditions, the CD error for L/S patterns driven by pulse energy fluctuations remains below 3 nm. For T2L patterns, the CD error induced by pulse energy instability can exceed 5 nm, thereby breaching typical exposure latitude (EL) specifications (i.e., <10% exposure latitude for the advanced node). To satisfy multi-pattern (1D/2D) requirements, pulse energy fluctuations should be maintained below 1%. Comparing the CD error trends for baseline bandwidths of 200 fm and 300 fm in Figure 9, this effect is principally attributable to coherence changes. As discussed in Section 2.2.2, temporal coherence is inversely related to bandwidth—narrower bandwidth yields stronger temporal coherence and more pronounced speckle. Equations (14)–(16) reveal that temporal degree of freedom *M* ∝ Δ*λ*; higher *M* diminishes field strength fluctuations, reducing CD errors. Therefore, increasing bandwidth during laser design mitigates coherence-related lithographic effects.

**Figure 10:**
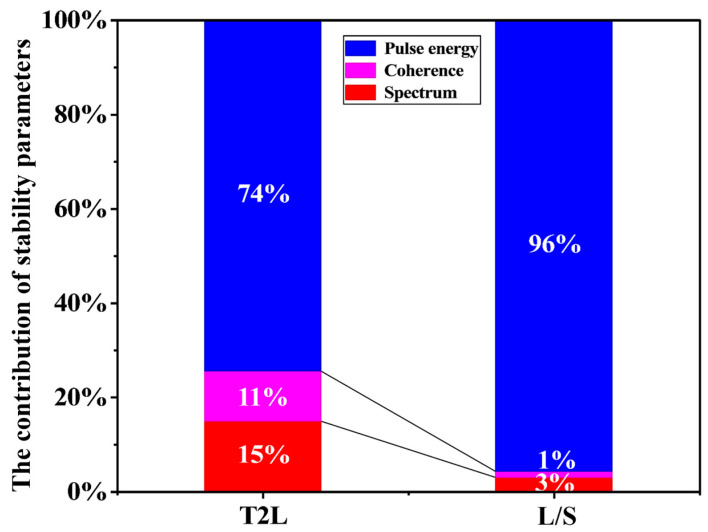
Contribution of light source parameter instabilities to CD error at Pitch = 90 nm.

As established in Section 2.2.1, spectral parameter variations ($\Delta_{\lambda ,\Delta \lambda }$) modify intensity distribution *I*(*x*, *y*), affecting local dose accumulation. Likewise, speckle effects (Δ*_c_*) from source coherence and pulse energy instability (Δ*_p_*) both contribute to dose fluctuations. Using a control of variables approach, we isolated the effects of spectral shape, coherence, and pulse energy stability on lithographic outcomes and quantified their relative contributions. Figure 10 summarizes the contributions of each source parameter instability to the total CD error for L/S and T2L patterns at Pitch = 90 nm. Results confirm that pulse energy instability dominates lithographic effects over bandwidth drift and coherence variations. Consequently, optimizing pulse energy stability should be prioritized in next-generation excimer laser development for advanced immersion lithography.

## 4. Conclusions

This study establishes a theoretical model quantifying lithographic impacts of stability parameter variations (spectral characteristics, coherence, and pulse energy) in excimer lasers for advanced-node immersion lithography. Simulations incorporating actual laser parameter shifts (μ, σ) during DUV lithography exposure were conducted using CD = 45 nm L/S and T2L test patterns. Spectral fluctuation effects ($\sigma_{\Delta \lambda }$, $\sigma_{\lambda }$) on aerial image intensity and NILS across multiple Pitches were investigated. Additionally, the photoresist model was included to evaluate the ADI-CD variations induced by pulse energy instability. Simulation results indicate that spectral variations cause CD errors within acceptable limits, whereas pulse energy instability exerts significantly stronger lithographic influence. For 45 nm linewidths, pulse energy-induced dose fluctuations must be confined to < 1%. We further observe that, for certain 2D patterns, careful tuning of spectral parameters (λ, ∆λ) can partially mitigate pulse energy effects. As technology nodes continue to shrink, laser parameter stability emerges as a critical factor for imaging fidelity, warranting deeper investigation. By elucidating the causality between light source instabilities and lithographic errors, this work provides a valuable reference for excimer laser development and process monitoring parameter optimization in advanced node manufacturing.

## Figures and Tables

**Table 1:** The experimental conditions.

Process Parameters	
Illimitation condition	cQuad: σ_out_ = 0.96, σ_inner_ = 0.65, blade angle = 20°
NA	1.35
Process	PTD
Mask type	Bright field and 6% attenuated PSM
Test pattern: Line/Space	
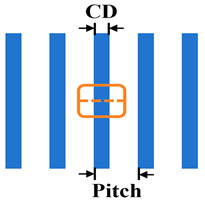	CD: 45 nm; Pitch: 90, 95, 100, 110, 120, 150, 200, 300, 500 nm
Test pattern: Tip to Line	
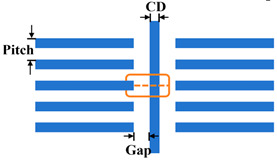	CD: 45 nm; Gap: 60, 65, 70 nm; Pitch: 90, 100, 110, 120, 150 nm;

**Table 2:** The excimer laser parameter stability conditions.

Laser Parameter Stability		
Bandwidth distribution change	$\mu_{\Delta \lambda }$	300, 200 fm
	$\sigma_{\Delta \lambda }$	0, 25, 50, 75, 100 fm
Central wavelength change	$\mu_{\lambda }$	193 nm
	$\sigma_{\lambda }$	0, 1, 2 pm
Pulse energy variation	$\mu_{E}$	102% (Assume that the deviation caused by the dose control of the light source system is 2%)
	$\sigma_{E}$	0.5, 1, 1.5, 2%

## Data Availability

The data underlying the results presented in this paper are not publicly available at this time but may be obtained from the authors upon reasonable request.

## Appendix A

Derivation of Formula (18):

Objective function:

(A1) $$O=K^{-1/2}\cdot x^{-1/2},\;K=\frac{\pi }{2\ln2}\cdot \frac{c∆T_{FWHM}}{\lambda^{2}},\;x=\Delta \lambda \;\;$$

The bandwidth Δ*λ* satisfies the normal distribution, i.e., $\Delta \lambda \sim N(\mu_{∆\lambda },\;\sigma_{\Delta \lambda }^{2})$. According to the statistical variance calculation formula, the variance Var[O] can be given by the expectation $E[O^{2}]$ and ${E[O]}^{2}$.

Step 1: Calculate $E[O^{2}]$

(A2) $$O^{2}=K^{-1}\cdot x^{-1},\;x=\Delta \lambda$$

At *x* = $\mu_{\Delta \lambda }$, perform Taylor expansion on the function $O^{2}$:

(A3) $$E[O^{2}]\approx K^{-1}[{\mu_{\Delta \lambda }}^{-1}-{\mu_{\Delta \lambda }}^{-2}E[x-\mu_{\Delta \lambda }]+{\mu_{\Delta \lambda }}^{-3}E[{\left(x-\mu_{\Delta \lambda }\right)}^{2}]$$

Substitute $E[x-\mu_{\Delta \lambda }]=E[∆\lambda ]-\mu_{\Delta \lambda }=0$ and $E[{\left(x-\mu_{\Delta \lambda }\right)}^{2}]=\sigma_{\Delta \lambda }^{2}$, and we receive

(A4) $$E[O^{2}]=K^{-1}{\mu_{\Delta \lambda }}^{-1}(1+\frac{\sigma_{\Delta \lambda }^{2}}{\mu_{\Delta \lambda }^{2}})$$

Step 2: Calculate ${E[O]}^{2}$

Squaring E[O], ignoring the effects of the higher-order term $\sigma_{\Delta \lambda }^{4}$ for small perturbations:

(A5) $${E[O]}^{2}\approx (K^{-1}{\mu_{\Delta \lambda }}^{-1})(1+\frac{3}{4}\frac{\sigma_{\Delta \lambda }^{2}}{\mu_{\Delta \lambda }^{2}})$$

Step 3: Calculate the variance

According to the formula $Var(O)=\;E[O^{2}]-{E[O]}^{2}$, we can obtain

(A6) $$Var(O)=\frac{1}{4}(K^{-1}{\mu_{\Delta \lambda }}^{-1})\cdot \frac{\sigma_{\Delta \lambda }^{2}}{\mu_{\Delta \lambda }^{2}}=\frac{1}{4}s_{0}^{2}{\left(\frac{\sigma_{\Delta \lambda }}{\mu_{\Delta \lambda }}\right)}^{2},\;s_{0}={\left(K\cdot \mu_{∆\lambda }\right)}^{1/2}=\sqrt{\frac{2\ln2}{\pi }}\frac{\lambda }{\sqrt{c\Delta T\mu_{\Delta \lambda }}}$$
